# Clinical outcomes of rTMS-augmented rehabilitation for post-stroke language and swallowing dysfunction: a sham-controlled prospective study with theory-informed interpretation

**DOI:** 10.3389/fneur.2026.1810365

**Published:** 2026-04-10

**Authors:** Li Sun, Jie Gao, Ying Wang, Yue Qiu, Qian Gu

**Affiliations:** Rehabilitation Medicine Department, Affiliated Hospital of Nantong University, Nantong, China

**Keywords:** aphasia, dysphagia, language recovery, neurorehabilitation, repetitive transcranial magnetic stimulation, stroke

## Abstract

**Background:**

Post-stroke language impairment and dysphagia frequently co-occur and can substantially hinder recovery, daily communication, and safe oral intake. Repetitive transcranial magnetic stimulation (rTMS) has been proposed as an adjunct to conventional rehabilitation, yet evidence on coordinated improvement across both domains remains limited, particularly in studies using clinically interpretable outcome measures.

**Methods:**

In this sham-controlled prospective study, 113 patients with post-stroke communication and swallowing impairment were enrolled and allocated to either active rTMS combined with conventional rehabilitation or sham stimulation plus the same rehabilitation program. The intervention lasted 2 weeks, with follow-up assessment after treatment completion. Clinical outcomes were evaluated at baseline, immediately after the intervention, and at follow-up using established measures of language performance, swallowing safety, and oral intake. Safety events and selected care-related indicators were also documented. Because no neurophysiological or neuroimaging markers were collected, mechanistic interpretation was limited to theory-informed inference based on observed clinical patterns.

**Results:**

Both groups improved over time. Compared with sham stimulation plus rehabilitation, the active rTMS group showed greater improvement in language-related and swallowing-related outcomes and maintained a more favorable recovery profile at follow-up. Directionally similar trends were observed across secondary clinical indicators, and no increase in safety risk was identified during the study period.

**Conclusions:**

rTMS administered before task-oriented rehabilitation was associated with improved short-term recovery in post-stroke language and swallowing function, with acceptable tolerability. These findings support further investigation of rTMS as an adjunct to multidisciplinary neurorehabilitation. Future randomized studies with longer follow-up and direct neurophysiological measures are needed to clarify mechanisms, durability of benefit, and patient-level heterogeneity in treatment response.

## 1 Introduction

Post-stroke communication and swallowing disorders are common complications that can substantially delay functional recovery and reduce quality of life after stroke (1). Dysphagia is associated with aspiration, pneumonia, malnutrition, dehydration, prolonged hospitalization, and poorer rehabilitation outcomes. Communication impairment is likewise frequent after stroke and may present as aphasia, dysarthria, or mixed clinical manifestations, each of which affects participation in therapy and daily interaction in different ways. In clinical practice, swallowing impairment often coexists with communication deficits, creating a more complex rehabilitation profile than either condition alone (2, 3).

Current post-stroke management generally emphasizes early screening, structured assessment, compensatory strategies, dietary modification, swallowing training, and speech-language rehabilitation delivered within a multidisciplinary framework. In this sense, contemporary care does not entirely treat each functional domain in isolation. However, the evidence base and many intervention protocols are still more commonly organized around single-domain outcomes, such as swallowing safety or language recovery alone. As a result, patients with concurrent impairments may receive parallel care without sufficient integration of therapeutic timing, intensity, and progression across domains (4). This remains clinically important because communication deficits can interfere with treatment participation and adherence, while dysphagia directly affects nutritional safety and medical stability.

Several challenges continue to limit recovery in patients with co-occurring deficits. First, stroke lesions vary considerably in location, extent, and network-level disruption. Language and swallowing functions involve partially overlapping but non-identical cortical and subcortical systems, which means that patients with superficially similar symptoms may respond differently to the same rehabilitation program (5, 6). Second, rehabilitation dosing is difficult to standardize in this population. Swallowing therapy often depends on repeated practice of safe sensorimotor patterns, whereas language rehabilitation relies on sustained cognitive engagement, task-specific repetition, and feedback. When both deficits are present, fatigue, reduced attention, impaired comprehension, and limited endurance may constrain treatment exposure and reduce the effectiveness of otherwise appropriate protocols. Third, variability in post-acute management, follow-up intensity, nutritional support, and prevention of pulmonary complications may further influence short-term and medium-term outcomes (7).

Against this background, non-invasive brain stimulation has attracted increasing interest as an adjunct to neurorehabilitation (8, 9). Repetitive transcranial magnetic stimulation (rTMS) is thought to modulate cortical excitability and influence interhemispheric or network-level activity patterns, potentially creating a more favorable context for experience-dependent rehabilitation. In post-stroke dysphagia, systematic reviews and network meta-analyses suggest that rTMS may improve swallowing-related outcomes, although uncertainty remains regarding optimal stimulation frequency, laterality, treatment dose, and target selection (10). In post-stroke aphasia, sham-controlled and increasingly individualized studies have also reported potential benefit, particularly when stimulation is paired with structured language therapy (11, 12).

Even so, several gaps remain. Much of the available literature addresses swallowing or language as separate targets, and fewer studies examine patients with coexisting impairments within an integrated rehabilitation pathway. In addition, many reports emphasize average treatment effects while giving less attention to how routinely collected clinical indicators behave across time, which measures are most informative for evaluating recovery, and how baseline heterogeneity may influence observed response patterns (13). These issues matter clinically, because patients with combined communication and swallowing deficits are often managed in settings where decisions rely primarily on practical bedside indicators rather than advanced physiological monitoring.

The present study therefore evaluated a structured protocol in which rTMS was delivered before conventional rehabilitation for post-stroke language and swallowing dysfunction. The study focused on clinically established indicators of language performance, swallowing safety, and oral intake across baseline, post-intervention, and follow-up assessment points. Because no neurophysiological or neuroimaging measures were collected, any interpretation regarding neural recovery in this study is necessarily theory-informed rather than directly demonstrated. Accordingly, the first objective was to examine whether rTMS combined with conventional rehabilitation was associated with greater short-term improvement than sham stimulation plus the same rehabilitation program. The second objective was to explore whether patterns across routinely collected clinical indicators could provide a clinically interpretable description of treatment response. Rather than claiming direct mechanistic proof, this study aimed to offer preliminary evidence that may inform the design, outcome selection, and methodological refinement of future randomized multicenter trials (14).

## 2 Methods

### 2.1 Study design, setting, and timeline

This study was designed as a prospective sham-controlled clinical study to evaluate the effectiveness and safety of repetitive transcranial magnetic stimulation (rTMS) combined with conventional rehabilitation in patients with post-stroke communication and swallowing impairment. The study was conducted in hospital departments affiliated with rehabilitation medicine. Consecutive patients meeting the eligibility criteria were screened during the recruitment period and underwent baseline evaluation before group assignment. Group assignment followed the predefined clinical study procedure used at the study site, and outcome assessors remained blinded to treatment condition throughout evaluation in order to reduce measurement bias.

The study comprised three stages: screening, intervention, and follow-up. During screening, investigators verified eligibility, explained the study procedures and possible risks, obtained written informed consent, and completed stimulation safety checks. The intervention period lasted 2 weeks, with 5 treatment sessions per week for a total of 10 sessions. On each treatment day, participants received either active rTMS or sham stimulation, followed by the same conventional speech-language and swallowing rehabilitation program delivered according to a standardized schedule. Outcome assessments were conducted at three predefined time points: baseline before the first treatment session, immediately after completion of the 2-week intervention, and 2 weeks after treatment completion. Baseline assessment was used to characterize the sample and examine between-group comparability. Post-intervention assessment was used to evaluate short-term treatment-associated change. Follow-up assessment was used to examine persistence of clinical improvement and to document delayed adverse events or interval clinical changes.

Safety monitoring was performed throughout the study period. Adverse events occurring during stimulation, rehabilitation, or follow-up were recorded in a structured manner together with any required management measures. A total of 113 participants entered the predefined efficacy and safety analysis set and were included in the final statistical analysis (15, 16).

### 2.2 Participants and eligibility criteria

**Figure 1:**
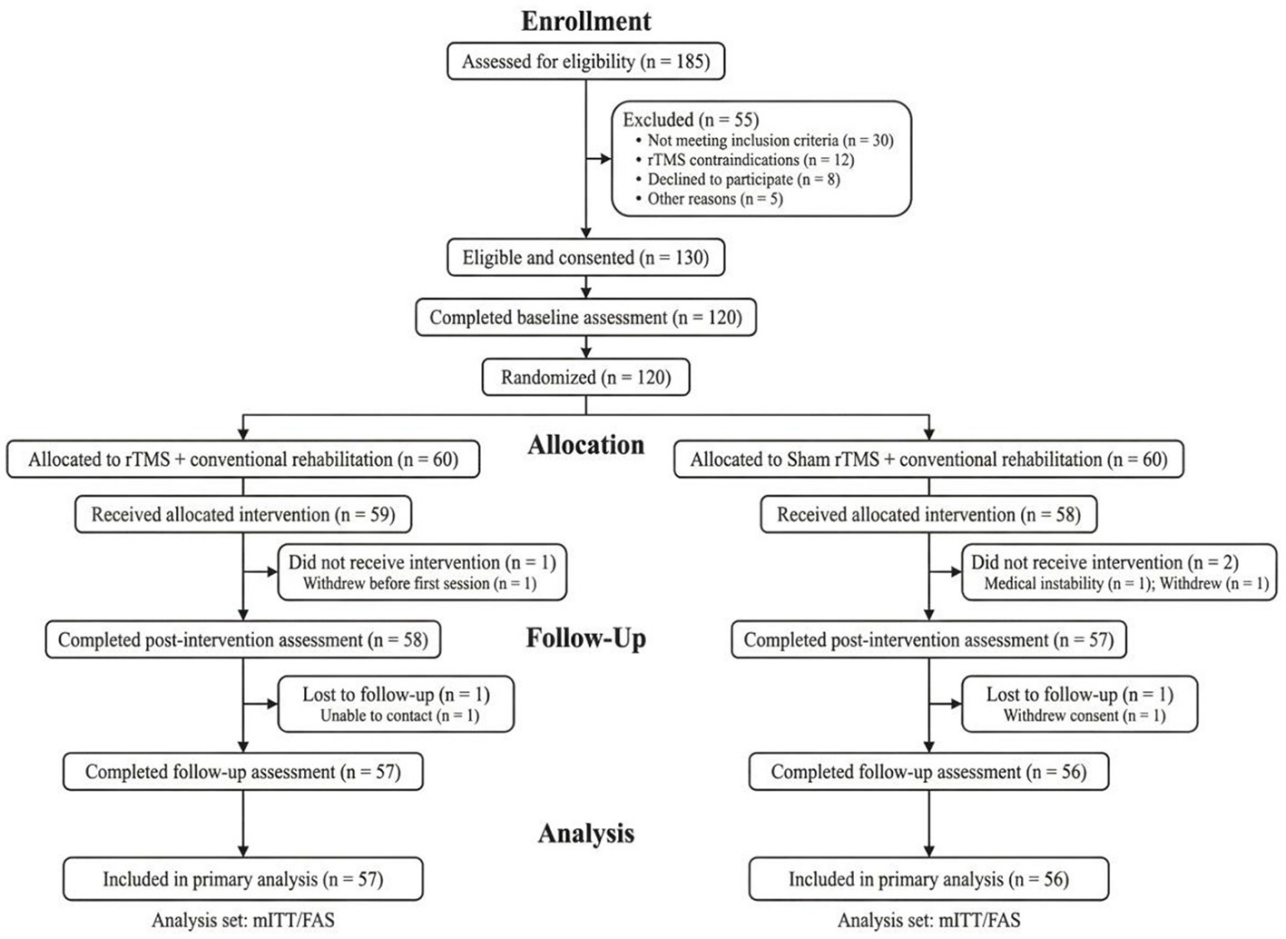
Flow diagram of participant screening, group allocation, follow-up, and analysis. The figure shows the number of participants screened for eligibility, excluded before enrollment, assigned to the active rTMS plus conventional rehabilitation group or the sham stimulation plus conventional rehabilitation group, followed through the intervention and follow-up periods, and included in the final analysis set. Reasons for exclusion, loss to follow-up, and non-completion are presented at each stage where applicable. The final analysis population comprised 113 participants.

Participants were recruited from consecutive patients evaluated in rehabilitation-related departments during the study period. All potentially eligible patients underwent standardized screening before enrollment. Written informed consent was obtained from participants or their legal representatives before collection of baseline data and study participation. The numbers screened, excluded, allocated, followed, and analyzed are shown in Figure 1.

Eligibility criteria were established to ensure diagnostic relevance, intervention feasibility, and stimulation safety. Participants were required to have a confirmed diagnosis of stroke based on clinical and imaging findings, together with both communication impairment and swallowing dysfunction after stroke. They were also required to be medically stable enough to tolerate rTMS and rehabilitation training and to have sufficient cooperation to complete the study assessments and treatment sessions. Communication impairment was defined on the basis of structured clinical evaluation performed by qualified rehabilitation or speech-language professionals. Because post-stroke communication disorders may include different clinical manifestations, the communication domain at enrollment could involve aphasia, dysarthria, or reduced functional communication performance documented in routine assessment. Swallowing dysfunction was defined using objective or semi-objective clinical evidence, including abnormal bedside swallowing screening, impaired swallowing scale results, or instrumental or imaging findings indicating reduced swallowing safety or efficiency.

Exclusion criteria were defined primarily on the basis of stimulation safety, feasibility of participation, and validity of assessment. Patients were excluded if they had contraindications to transcranial magnetic stimulation, including epilepsy, markedly increased seizure risk, or implanted metallic or electronic devices that could be affected by magnetic stimulation. Patients with severe disturbance of consciousness or cognitive impairment preventing reliable evaluation or active participation in treatment were also excluded. Additional exclusion criteria included unstable cardiopulmonary disease or other serious medical conditions that could compromise intervention safety, progressive neurological disease, and severe psychiatric or behavioral disturbance expected to substantially interfere with adherence, follow-up, or outcome assessment.

Because the study included patients with heterogeneous post-stroke communication profiles, the communication domain was characterized clinically at baseline, whereas the primary language-related outcome measure was analyzed separately in the outcome section. This distinction was made to avoid conflating broader communication impairment with aphasia-specific assessment.

### 2.3 Intervention protocols: rTMS and conventional rehabilitation

The intervention protocol was structured to reflect a clinically feasible combined neurorehabilitation pathway. All participants completed baseline assessment before the start of treatment. The intervention course lasted 2 weeks, with 10 clinic-based sessions administered on weekdays. Both groups received the same conventional rehabilitation schedule and dose, and the only between-group difference was whether active or sham rTMS was delivered before rehabilitation. This design was intended to minimize confounding related to training intensity and therapist exposure.

rTMS was delivered using a clinical-grade figure-of-eight coil system. The stimulation site was the left ventrolateral primary motor cortex, localized using a standard scalp-based approach supported by motor hotspot confirmation and resting motor threshold testing from the abductor pollicis brevis. In the active stimulation group, high-frequency rTMS was delivered at 10 Hz and 90% resting motor threshold, with 5-s trains, 25-s inter-train intervals, 40 trains per session, and 2,000 pulses per session, yielding a cumulative dose of 20,000 pulses over the 10-session treatment course. Each stimulation session lasted approximately 20 min.

In the sham condition, the same anatomical targeting procedure, apparent device settings, treatment schedule, and session timing were used, but cortical stimulation was minimized by the sham setup. This approach was used to preserve procedural similarity between groups while reducing active neuromodulatory effects. All stimulation sessions were delivered by trained personnel who completed pre-session safety checks. Treatment could be paused or terminated if adverse symptoms occurred or if continuation was judged clinically inappropriate.

Immediately after stimulation, participants in both groups received the same conventional rehabilitation program. Rehabilitation generally began within 10–15 min after completion of active or sham stimulation in order to preserve temporal continuity between neuromodulation and task-oriented practice. The rehabilitation program consisted of two standardized components delivered on the same day. The speech-language component lasted 30 min per session and included exercises targeting comprehension, expression, naming, repetition, articulation, respiratory-phonatory coordination, and functional communication tasks as clinically appropriate. The swallowing component also lasted 30 min per session and included individualized dysphagia rehabilitation measures such as oropharyngeal muscle exercises, sensory stimulation, task-specific swallowing maneuvers, posture adjustment, bolus modification strategies, and safe feeding education. Accordingly, each clinic-based rehabilitation session lasted 60 min, corresponding to a cumulative in-clinic rehabilitation dose of 600 min across the 10-session intervention period.

To support continuity of practice outside the clinic, all participants received the same structured home-based training plan, consisting of approximately 20 min per day for 14 days. The home program included guided communication exercises and safe swallowing practice, with completion recorded in a daily log. The cumulative planned home-practice dose was 280 min. Treatment fidelity was monitored using attendance records, therapist checklists, and review of home-practice logs. Session completion and module delivery were verified throughout the intervention period. Information on concomitant treatments or care factors that could influence outcomes was collected during follow-up and was incorporated into subsequent interpretation and sensitivity analysis. Safety monitoring was applied in both groups throughout the intervention. Pre- and post-session checks included symptoms such as headache, dizziness, fatigue, nausea, or mood change, and additional vital-sign assessment was performed when clinically indicated. Emergency procedures for seizure management were available throughout the treatment period. The detailed treatment schedule and dose parameters are summarized in Table 1.

**Table 1:** rTMS and conventional rehabilitation protocol details and treatment dose.

Component	Active rTMS + Conventional rehabilitation	Sham rTMS + Conventional rehabilitation
Study phase	Intervention phase (2 weeks)	Intervention phase (2 weeks)
Schedule	5 sessions/week for 2 weeks (total 10 sessions)	5 sessions/week for 2 weeks (total 10 sessions)
rTMS device	Figure-of-eight coil stimulator (clinical-grade)	Sham coil or identical coil angled to minimize effective cortical stimulation
Target region	Left primary motor cortex (M1) swallowing–speech motor representation (ventrolateral M1)	Same anatomical target procedure as active group
Localization method	10–20 EEG system with motor hotspot confirmation; RMT determined from abductor pollicis brevis (APB)	Same localization and RMT determination
Stimulation pattern	High-frequency rTMS	Sham stimulation (auditory click and scalp sensation mimicked)
Frequency	10 Hz	10 Hz (sham output)
Intensity	90% resting motor threshold (RMT)	90% RMT displayed, sham output with negligible induced field
Train structure	5 s on, 25 s inter-train interval	Same timing
Number of trains per session	40	40
Pulses per train	50	50
Pulses per session	2,000	2,000 (sham)
rTMS duration per session	~20 min	~20 min
Cumulative rTMS pulses	20,000 pulses over 10 sessions	20,000 pulses over 10 sessions (sham)
Timing relative to rehabilitation	Conventional rehabilitation started within 10–15 min after rTMS	Conventional rehabilitation started within 10–15 min after sham
Conventional rehabilitation (core modules)	Standardized speech-language therapy + standardized dysphagia rehabilitation	Identical standardized speech-language therapy + standardized dysphagia rehabilitation
Speech-language therapy (per session)	30 min/session (naming, comprehension, repetition, articulation, respiratory–phonatory coordination, functional communication tasks)	30 min/session (same content)
Dysphagia rehabilitation (per session)	30 min/session (oropharyngeal muscle exercises, sensory stimulation, effortful swallow/Mendelsohn as appropriate, posture and bolus modification training, safe feeding education)	30 min/session (same content)
Rehabilitation dose (per session)	60 min/session	60 min/session
Cumulative rehabilitation dose	600 min over 10 sessions	600 min over 10 sessions
Home program	20 min/day for 14 days (structured exercises and safe swallowing practice log)	20 min/day for 14 days (same log)
Home program cumulative dose	280 min	280 min
Total combined dose (clinic + home)	880 min (600 clinic + 280 home)	880 min (600 clinic + 280 home)
Fidelity and adherence monitoring	Attendance recorded each session; therapist checklist for module completion; home log reviewed twice weekly	Same monitoring
Safety monitoring	Pre/post-session adverse event checklist (headache, dizziness, fatigue, nausea, mood change); vitals if clinically indicated; seizure emergency protocol available	Same monitoring

### 2.4 Target localization and intervention workflow

To improve procedural consistency and reproducibility, target localization and treatment delivery were implemented according to a standardized workflow. The intervention sequence was fixed across participants so that stimulation was always delivered before task-oriented rehabilitation. This order was chosen on the basis of the working assumption that transient post-stimulation changes in cortical excitability may increase the responsiveness of subsequent behavioral training, although this mechanism was not directly measured in the present study.

**Figure 2:**
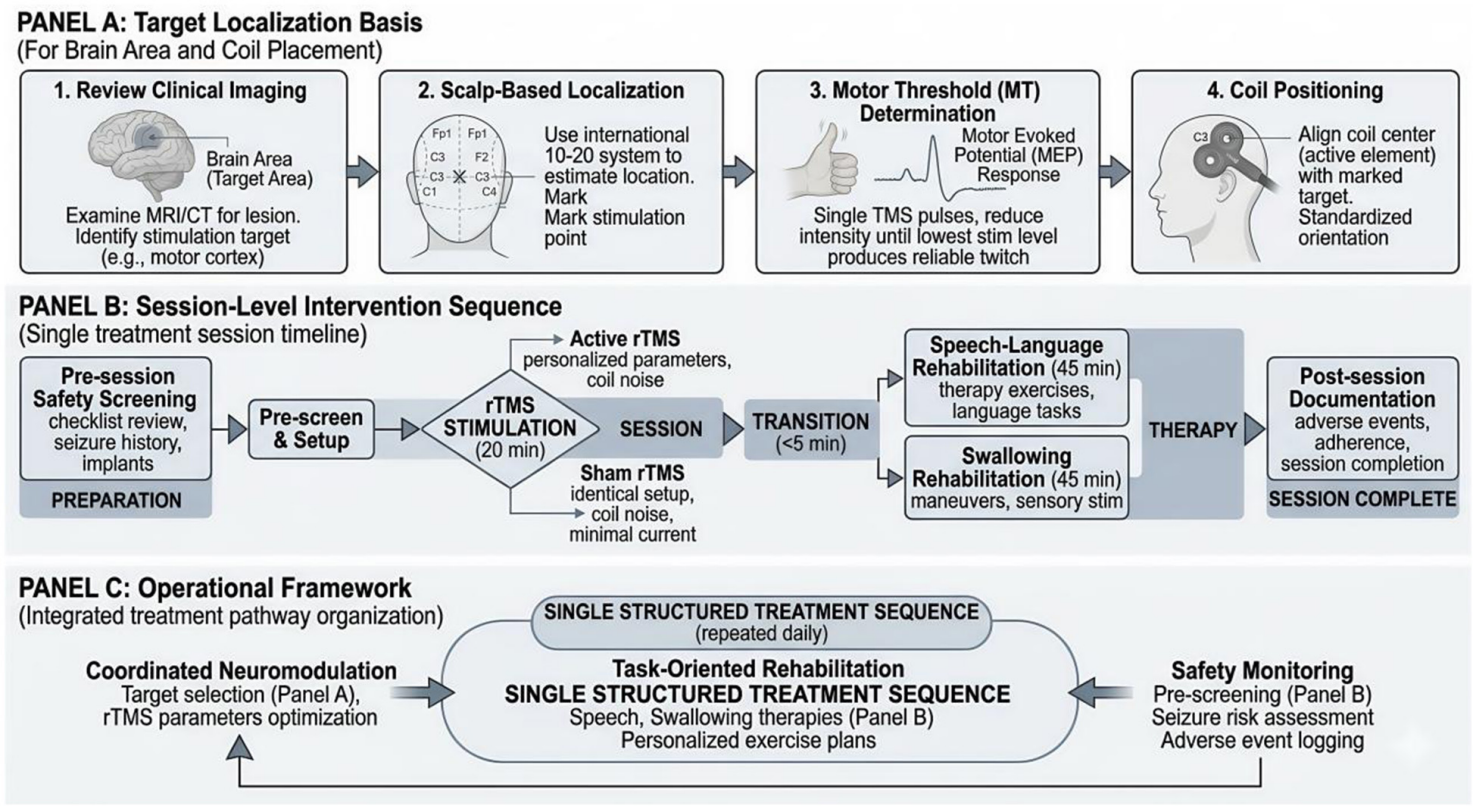
Standardized workflow for target localization and integrated rTMS-augmented rehabilitation in post-stroke communication and swallowing impairment. Panel **(A)** summarizes the practical basis for target localization, including review of available clinical imaging, scalp-based localization, motor threshold determination, and coil positioning. Panel **(B)** illustrates the session-level intervention sequence, including pre-session safety screening, active or sham stimulation, immediate transition to speech-language and swallowing rehabilitation, and post-session documentation. Panel **(C)** outlines the operational framework of the integrated pathway, showing how neuromodulation, task-oriented rehabilitation, and safety monitoring were coordinated within one treatment sequence. Panel **(C)** is presented as a procedural framework to support reproducibility and clinical interpretation and should not be interpreted as direct evidence of a verified biological mechanism.

As shown in Figure 2, the workflow comprised three complementary components. Panel A summarizes the practical basis for target selection, including review of available clinical imaging, scalp-based localization, motor threshold determination, and coil positioning. Panel B presents the session-level procedure, including pre-session safety screening, active or sham stimulation, immediate transition to speech-language and swallowing rehabilitation, and post-session documentation. Panel C outlines the operational rationale of the integrated pathway, namely the structured coordination of neuromodulation, task-oriented rehabilitation, and safety monitoring within one treatment sequence. In this study, Panel C serves as a procedural framework rather than as direct evidence of a verified biological mechanism.

**Figure 3:**
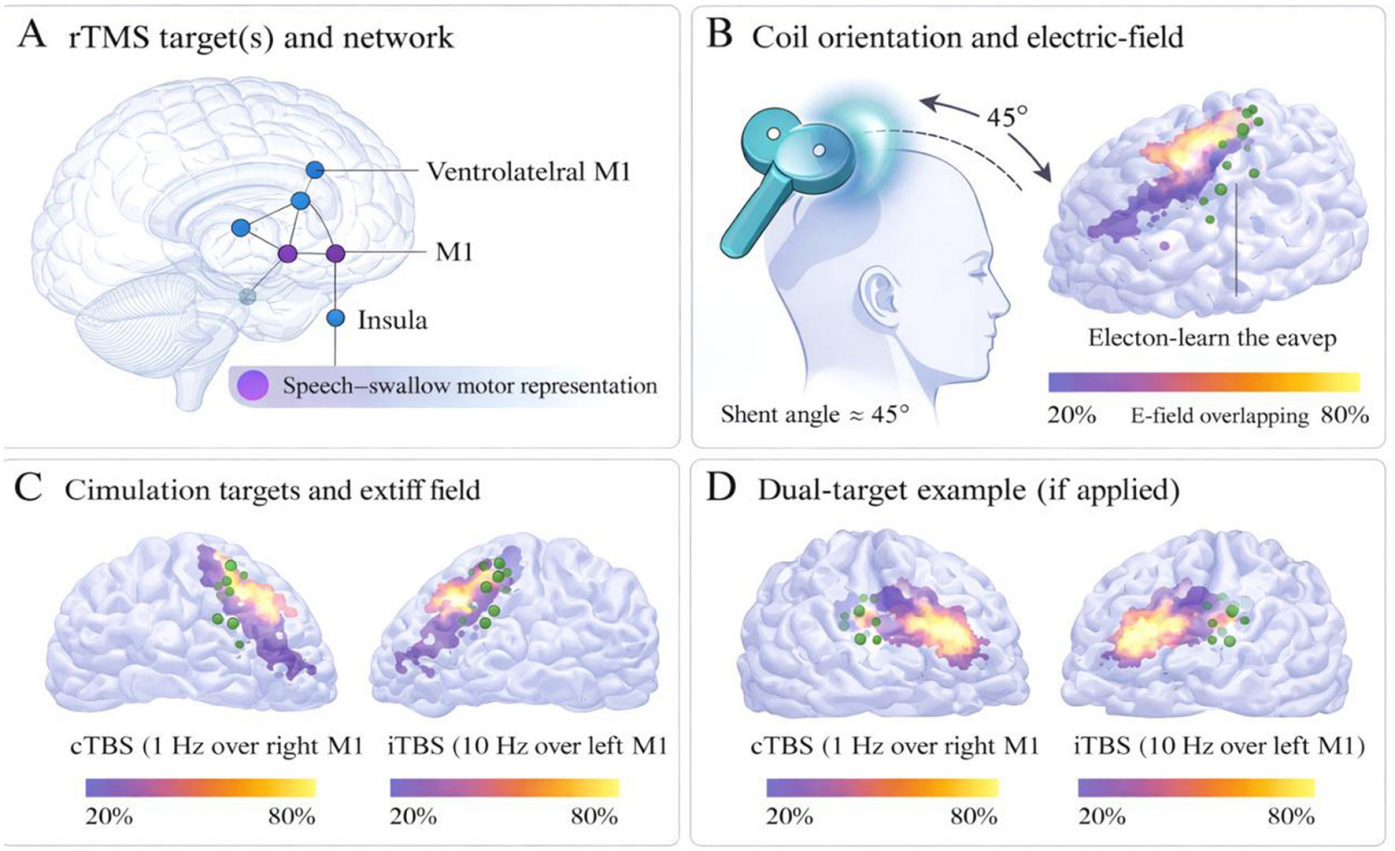
Representative stimulation target localization and coil orientation in the integrated rehabilitation protocol. **(A)** Shows the candidate cortical region used for stimulation targeting within the integrated rehabilitation protocol. **(B)** Illustrates representative coil orientation and the corresponding stimulation geometry. **(C)** Shows an example of a single-target stimulation configuration, and **(D)** shows an example of an alternative target arrangement when clinically applicable. These panels are provided to clarify target placement, coil positioning, and procedural consistency across sessions. They are intended as technical reference illustrations and not as direct evidence of treatment-related neural mechanism.

For target localization, available clinical imaging and neurological findings were reviewed to understand lesion context and relevant functional anatomy. Initial target selection was guided by conventional scalp-based localization procedures, with additional navigation support used when available to improve positional consistency. Stimulation intensity was individualized according to resting motor threshold testing. Under standardized positioning procedures, coil placement and orientation were adjusted according to participant anatomy in order to reduce spatial deviation across sessions. The anatomical distribution of stimulation targets and representative coil orientation are illustrated in Figure 3. These visual materials are provided to clarify target placement and physical setup rather than to imply direct measurement of neural recovery mechanisms.

On each treatment day, stimulation was administered first, and conventional rehabilitation was initiated within a comparable interval after stimulation. This sequence was documented using therapist checklists and session records. Tolerability and adverse events were assessed before and after each session. If adverse symptoms, warning signs, or poor tolerance emerged, the procedure was modified, interrupted, or terminated according to the predefined safety procedure to ensure participant safety throughout the treatment course.

### 2.5 Outcomes and assessment schedule

Outcome selection was designed to reflect the dual clinical focus of the study while preserving conceptual clarity regarding the distinction between broader communication impairment at enrollment and specific assessment domains used in analysis. Because the enrolled sample included heterogeneous post-stroke communication profiles, communication impairment was characterized clinically at baseline, whereas quantitative analysis was anchored to predefined language-related and swallowing-related instruments.

The primary outcomes were functional oral intake, swallowing safety, and language-related impairment severity. Functional oral intake was assessed using the Functional Oral Intake Scale (FOIS), swallowing safety was assessed using the Penetration–Aspiration Scale (PAS) based on instrumental swallowing evaluation when available, and language-related impairment severity was assessed using the Western Aphasia Battery–Revised Aphasia Quotient (WAB-R AQ) in participants who were eligible for aphasia-oriented language assessment. This distinction was made because the enrolled cohort could include broader post-stroke communication impairment, such as aphasia, dysarthria, or reduced functional communication, whereas WAB-R AQ specifically reflects language impairment rather than the entire spectrum of speech-related dysfunction. These primary outcomes were selected because they represent three clinically important dimensions of post-stroke recovery, namely oral intake, airway safety, and language-related communication performance.

Secondary outcomes were included to capture broader clinical relevance. These comprised functional communication, assessed by the Communication Effectiveness Index (CETI); bedside dysphagia severity, assessed by the Mann Assessment of Swallowing Ability (MASA); dysphagia outcome level, assessed by the Dysphagia Outcome and Severity Scale (DOSS); swallowing-related quality of life, assessed by SWAL-QOL; feeding-tube dependence; aspiration pneumonia during the study period; global disability, assessed by the modified Rankin Scale (mRS); activities of daily living, assessed by the Barthel Index (BI); and neurological deficit severity, assessed by the National Institutes of Health Stroke Scale (NIHSS). Process-related indicators included session attendance and home-program completion, and safety outcomes included adverse events together with session-level discomfort ratings.

Assessments were conducted at three predefined time points. Baseline assessment was performed before the first treatment session and was used to characterize the sample and establish between-group comparability. Post-intervention assessment was conducted at the end of the 2-week treatment period and was used to evaluate short-term treatment-associated change. Follow-up assessment was conducted 2 weeks after treatment completion and was used to examine whether observed gains were maintained and whether delayed adverse events or interval clinical events occurred. Adverse events were monitored continuously from screening through the end of follow-up.

Detailed definitions of endpoints, assessment tools, score ranges, direction of favorable change, assessment time points, and study-defined responder thresholds are summarized in Table 2. Because well-established minimal clinically important differences were not available for several measures used in this study, responder thresholds were defined a priori for descriptive and exploratory interpretation and should not be interpreted as universally established clinical cutoffs.

**Table 2:** Outcome definitions, assessment tools, score ranges, direction of favorable change, assessment time points, and study-defined responder thresholds.

Outcome domain	Endpoint (type)	Assessment tool (abbrev.)	Score range/unit	Direction (better)	Assessment time points	Study-defined responder threshold/reporting approach
Swallowing function	Functional oral intake (Primary)	Functional Oral Intake Scale (FOIS)	1–7 levels	Higher	T0, T1, T2	Responder: ≥1-level increase from T0
Swallowing safety	Airway invasion severity (Primary)	Penetration–Aspiration Scale (PAS) from VFSS/FEES	1–8	Lower	T0, T1, T2	Responder: ≥2-point decrease from T0
Language-related impairment	Global aphasia severity (Primary language-related outcome)	Western Aphasia Battery–Revised Aphasia Quotient (WAB-R AQ)	0–100	Higher	T0, T1, T2	Responder: ≥5-point increase from T0
Communication participation	Functional communication (Secondary)	Communication Effectiveness Index (CETI)	0–100	Higher	T0, T1, T2	Responder: ≥10-point increase from T0
Dysphagia clinical severity	Bedside dysphagia severity (Secondary)	Mann Assessment of Swallowing Ability (MASA)	0–200	Higher	T0, T1, T2	Responder: ≥10-point increase from T0
Diet/feeding safety	Dysphagia outcome level (Secondary)	Dysphagia Outcome and Severity Scale (DOSS)	1–7 levels	Higher	T0, T1, T2	Responder: ≥1-level increase from T0
Swallow-related quality of life	Swallow-related quality of life (Secondary)	SWAL-QOL (total score)	0–100	Higher	T0, T2	Responder: ≥10-point increase from T0
Nutrition/route of feeding	Feeding tube dependence (Secondary clinical outcome)	Clinical record	Yes/No	No tube	T0, T1, T2	Report proportion with tube removal by T2 among those with tube feeding at T0
Pulmonary complications	Aspiration pneumonia (Secondary clinical event)	Clinical diagnosis supported by imaging and laboratory findings	Event/Yes–No	Fewer events	Continuous during study; summarized at T1 and T2	Report incidence and risk estimate
Global disability	Functional status (Secondary)	Modified Rankin Scale (mRS)	0–6	Lower	T0, T2	Responder: ≥1-grade decrease from T0
Activities of daily living	Independence (Secondary)	Barthel Index (BI)	0–100	Higher	T0, T2	Responder: ≥10-point increase from T0
Neurological deficit	Stroke severity (Covariate/supportive secondary outcome)	NIH Stroke Scale (NIHSS)	0–42	Lower	T0, T1, T2	Responder: ≥2-point decrease from T0
Treatment adherence	Session attendance (Process indicator)	Attendance log	0–10 sessions	Higher	Throughout	Adequate adherence: ≥8/10 sessions completed
Home program adherence	Home practice completion (Process indicator)	Patient/caregiver log	% days completed	Higher	Throughout	Adequate adherence: ≥70% of days completed
Safety/tolerability	Adverse events (Safety)	AE checklist plus clinician assessment	Count; severity grade	Fewer/milder	Each session; summarized at T1 and T2	Report incidence, seriousness, and relatedness
Safety/tolerability	Discomfort during stimulation (Safety)	Numeric rating scale (NRS)	0–10	Lower	Each session	Report severity and prespecified stopping rule for persistent severe discomfort

### 2.6 Safety monitoring and adverse event reporting

Safety evaluation was integrated throughout screening, intervention, and follow-up. Before enrollment, all participants underwent screening for contraindications to transcranial magnetic stimulation, including seizure history, increased seizure risk, metallic or electronic implants, recent neurosurgical procedures, and other conditions potentially affecting stimulation safety. Before each treatment session, a brief clinical safety review was performed to identify transient conditions that could increase risk, such as sleep deprivation, fever, blood pressure instability, or acute physical discomfort. When clinically indicated, treatment was postponed, modified, or discontinued.

Adverse events were documented using a structured checklist applied before and after treatment sessions. Monitored events included headache, scalp discomfort or pain, dizziness, nausea, fatigue, emotional changes, transient neurological symptoms, treatment-related coughing, suspected aspiration, and respiratory changes during training. Each event was recorded with respect to timing, duration, severity, management, outcome, and possible relationship to the intervention. Events were classified as serious or non-serious according to clinical severity and consequence. When predefined stopping criteria were met or a serious adverse event occurred, the intervention was terminated immediately and appropriate emergency procedures were initiated. Expert consultation was obtained when required, and notification procedures followed institutional ethical and regulatory requirements.

Safety surveillance was continued during follow-up to capture delayed adverse reactions and interval swallowing-related clinical events. Follow-up safety information was obtained through scheduled reassessment and, when necessary, telephone contact. This approach was used to provide a more complete description of overall tolerability and clinical risk during the study period.

### 2.7 Data quality, blinding, and statistical analysis including clinically interpretable analysis

To ensure data quality, evaluators and therapists received standardized training before study initiation, and uniform operating manuals, case-report forms, and documentation procedures were implemented. Assessments were conducted using fixed sequences and standardized instructions. Whenever feasible, the same assessor evaluated the primary outcomes for a given participant to reduce inter-rater variability. Data entry was completed independently by two trained staff members, followed by logical validation and cross-checking against source documents. Missing or inconsistent values were reviewed according to predefined data-management rules. Attendance records, home-practice logs, protocol deviations, and relevant concomitant treatments were documented throughout the study and were considered during interpretation and sensitivity analysis.

Outcome assessors remained blinded to treatment allocation throughout evaluation. Participant blinding was maintained through sham stimulation procedures designed to approximate the operational experience of active treatment as closely as possible under clinical conditions. Personnel who delivered stimulation followed protocol requirements and did not participate in outcome assessment. They were also instructed not to disclose treatment allocation to evaluators or participants during the study period.

All statistical analyses were performed within the predefined analysis population using two-sided tests, with a significance level of 0.05. Continuous outcomes measured repeatedly across time, including WAB-R AQ, CETI, MASA, SWAL-QOL, BI, NIHSS, and session-level discomfort scores, were analyzed using linear mixed-effects models with fixed effects for group, time, and the group-by-time interaction, together with a participant-level random intercept to account for within-subject correlation. For each model, the corresponding baseline value was incorporated as an adjustment term when appropriate. Ordinal repeated outcomes, including FOIS, PAS, DOSS, and mRS, were analyzed using generalized linear mixed models with a cumulative logit link. Binary clinical outcomes, such as aspiration pneumonia and feeding-tube dependence, were analyzed using generalized estimating equations or mixed-effects logistic regression according to the distribution and frequency of observed events. Estimated between-group differences were reported together with 95% confidence intervals and effect-size measures where applicable.

Prespecified covariates included age, sex, time since stroke, baseline NIHSS score, lesion laterality when available, and the baseline value of the corresponding outcome measure. These variables were selected because of their potential association with neurological recovery, swallowing safety, and response to rehabilitation. When substantial baseline imbalance was not observed, the same covariate-adjusted framework was retained to maintain analytic consistency across models.

Because three primary outcomes were prespecified, multiplicity across the primary endpoint family was addressed using the Holm procedure. Secondary outcomes were interpreted as supportive and exploratory and were therefore not used for confirmatory inference. This distinction was maintained throughout the Results and Discussion to avoid overinterpretation of secondary analyses.

Missing data were first examined descriptively to determine their extent and pattern. Primary analyses were based on mixed-effects models, which use all available repeated observations under a missing-at-random assumption and therefore reduce the loss of information caused by incomplete follow-up. In addition, sensitivity analyses were conducted using multiple imputation by chained equations with 20 imputed datasets for outcomes or covariates with missing follow-up values. The imputation model included treatment group, time point, baseline severity, age, sex, time since stroke, and the relevant observed outcome values. Results from the imputed datasets were compared with complete-case analyses to examine the robustness of the findings.

To address the study objective of interpretability, a clinically interpretable response analysis was performed in parallel with the model-based analyses. In this analysis, each participant was assigned an integrated response score ranging from 0 to 3 according to the number of primary domains in which the prespecified responder threshold was achieved. One point was assigned for a FOIS increase of at least one level from baseline, one point was assigned for a PAS decrease of at least two points from baseline, and one point was assigned for a WAB-R AQ increase of at least five points from baseline. For participants in whom aphasia-oriented assessment was not applicable, the language-related response component was defined using a CETI increase of at least 10 points from baseline. Higher integrated response scores therefore indicated broader multidomain improvement. This score was used for the clinically interpretable analysis described in the Results section and was analyzed descriptively and, where appropriate, with ordinal regression models to compare response distribution between groups across time.

To further characterize heterogeneity of treatment response, prespecified interaction terms were examined, including baseline severity by group and time since stroke by group. Incremental explanatory contribution was evaluated using nested-model comparison metrics, including changes in Akaike information criterion and likelihood-based fit indices, to identify which routinely collected clinical indicators most consistently accounted for treatment-associated improvement. In this study, such analyses were intended to provide a clinically interpretable account of response patterns rather than to establish direct neural mechanisms. Accordingly, any discussion linking observed outcome patterns to neuroplasticity models was treated as theory-informed inference and not as evidence derived from direct neurophysiological or neuroimaging measurement in the present dataset.

## 3 Results

### 3.1 Baseline characteristics and lesion distribution

A total of 113 participants were included in the full analysis set, comprising 57 individuals in the rTMS plus conventional rehabilitation group and 56 in the sham stimulation plus conventional rehabilitation group. Overall, baseline demographic and clinical characteristics were comparable between groups. The mean age was 59.3 ± 12.7 years in the rTMS group and 58.1 ± 13.4 years in the sham group. Female participants accounted for 42.1% and 44.6% of the two groups, respectively. Time since stroke was also similar between groups, with a median of 24 days in the rTMS group and 26 days in the sham group. Baseline neurological severity and functional status were closely matched, with a median NIHSS score of 7 in both groups, a median modified Rankin Scale score of 4 in both groups, and mean Barthel Index scores of 41.7 ± 18.9 and 43.1 ± 19.4, respectively.

Baseline speech-language and swallowing status was likewise comparable between groups. Aphasia was present in 71.9% of the rTMS group and 69.6% of the sham group, whereas dysarthria was documented in 54.4% and 48.2%, respectively. Mean WAB-R Aphasia Quotient scores were 55.6 ± 14.8 in the rTMS group and 54.6 ± 15.5 in the sham group. Median FOIS level was 3 in both groups, and median PAS score was 6 in both groups. Severe airway invasion, defined as PAS 6–8, was observed in 68.4% of the rTMS group and 69.6% of the sham group. Feeding tube use at baseline was present in 31.6% and 28.6% of participants, respectively. No statistically significant between-group differences were identified across the baseline variables shown in Table 3.

**Table 3:** Baseline demographic and clinical characteristics of the full analysis set.

Variable	Total n = 113	rTMS + Rehab n = 57	Sham + Rehab n = 56	*P-value*
Age, years, mean ± SD	58.7 ± 13.0	59.3 ± 12.7	58.1 ± 13.4	0.623
Female sex, *n* (%)	49 (43.4)	24 (42.1)	25 (44.6)	0.789
Body mass index, kg/m^2^, mean ± SD	23.9 ± 3.7	23.8 ± 3.6	24.1 ± 3.8	0.663
Years of education, median (IQR)	9 (6–12)	9 (6–12)	10 (6–12)	0.481
Right-handed, *n* (%)	102 (90.3)	52 (91.2)	50 (89.3)	0.867
Time since stroke, days, median (IQR)	25 (15–42)	24 (14–41)	26 (15–44)	0.534
Time since stroke category, *n* (%)				0.812
Acute (< 14 days)	21 (18.6)	11 (19.3)	10 (17.9)	
Subacute (14–90 days)	81 (71.7)	41 (71.9)	40 (71.4)	
Chronic (>90 days)	11 (9.7)	5 (8.8)	6 (10.7)	
Etiology, *n* (%)				0.941
Ischemic stroke	86 (76.1)	43 (75.4)	43 (76.8)	
Hemorrhagic stroke	27 (23.9)	14 (24.6)	13 (23.2)	
Lesion side, *n* (%)				0.736
Left hemisphere	56 (49.6)	29 (50.9)	27 (48.2)	
Right hemisphere	39 (34.5)	20 (35.1)	19 (33.9)	
Bilateral/brainstem	18 (15.9)	8 (14.0)	10 (17.9)	
Lesion location, *n* (%)				0.764
Cortical	40 (35.4)	21 (36.8)	19 (33.9)	
Subcortical	50 (44.2)	24 (42.1)	26 (46.4)	
Cortico-subcortical (mixed)	23 (20.4)	12 (21.1)	11 (19.6)	
NIHSS score, median (IQR)	7 (4–10)	7 (4–10)	7 (4–11)	0.709
Modified Rankin Scale, median (IQR)	4 (3–4)	4 (3–4)	4 (3–4)	0.892
Barthel index, mean ± SD	42.4 ± 19.1	41.7 ± 18.9	43.1 ± 19.4	0.742
Aphasia present, *n* (%)	80 (70.8)	41 (71.9)	39 (69.6)	0.931
Dysarthria present, *n* (%)	58 (51.3)	31 (54.4)	27 (48.2)	0.643
WAB-R Aphasia Quotient, mean ± SD	55.1 ± 15.1	55.6 ± 14.8	54.6 ± 15.5	0.721
Moderate-to-severe aphasia (WAB-R AQ < 60), *n* (%)	63 (55.8)	32 (56.1)	31 (55.4)	0.919
FOIS level, median (IQR)	3 (2–4)	3 (2–4)	3 (2–4)	0.903
Feeding tube at baseline, *n* (%)	34 (30.1)	18 (31.6)	16 (28.6)	0.781
PAS score, median (IQR)	6 (5–7)	6 (5–7)	6 (5–7)	0.837
Severe airway invasion (PAS 6–8), *n* (%)	78 (69.0)	39 (68.4)	39 (69.6)	0.742
MASA total score, mean ± SD	145.8 ± 21.6	146.2 ± 21.3	145.3 ± 22.0	0.831
CETI, mean ± SD	36.9 ± 14.7	37.4 ± 14.5	36.3 ± 15.0	0.694
DOSS level, median (IQR)	3 (2–4)	3 (2–4)	3 (2–4)	0.886
SWAL-QOL total, mean ± SD	54.1 ± 12.8	54.3 ± 12.7	54.0 ± 12.9	0.917
Serum albumin, g/L, mean ± SD	38.7 ± 4.6	38.5 ± 4.7	38.9 ± 4.5	0.631
Aspiration pneumonia within 30 days before enrollment, *n* (%)	23 (20.4)	12 (21.1)	11 (19.6)	0.913
Hypertension, *n* (%)	60 (53.1)	29 (50.9)	31 (55.4)	0.563
Diabetes mellitus, *n* (%)	25 (22.1)	13 (22.8)	12 (21.4)	0.921
Atrial fibrillation, *n* (%)	15 (13.3)	8 (14.0)	7 (12.5)	0.823
Hyperlipidemia, *n* (%)	37 (32.7)	19 (33.3)	18 (32.1)	0.887
COPD/asthma, *n* (%)	11 (9.7)	5 (8.8)	6 (10.7)	0.657
Current smoker, *n* (%)	26 (23.0)	14 (24.6)	12 (21.4)	0.593
Current alcohol use, *n* (%)	19 (16.8)	10 (17.5)	9 (16.1)	0.883
Antithrombotic therapy, *n* (%)	76 (67.3)	39 (68.4)	37 (66.1)	0.927
Statin use, *n* (%)	68 (60.2)	33 (57.9)	35 (62.5)	0.487
ACEI/ARB use, *n* (%)	53 (46.9)	26 (45.6)	27 (48.2)	0.769

**Figure 4:**
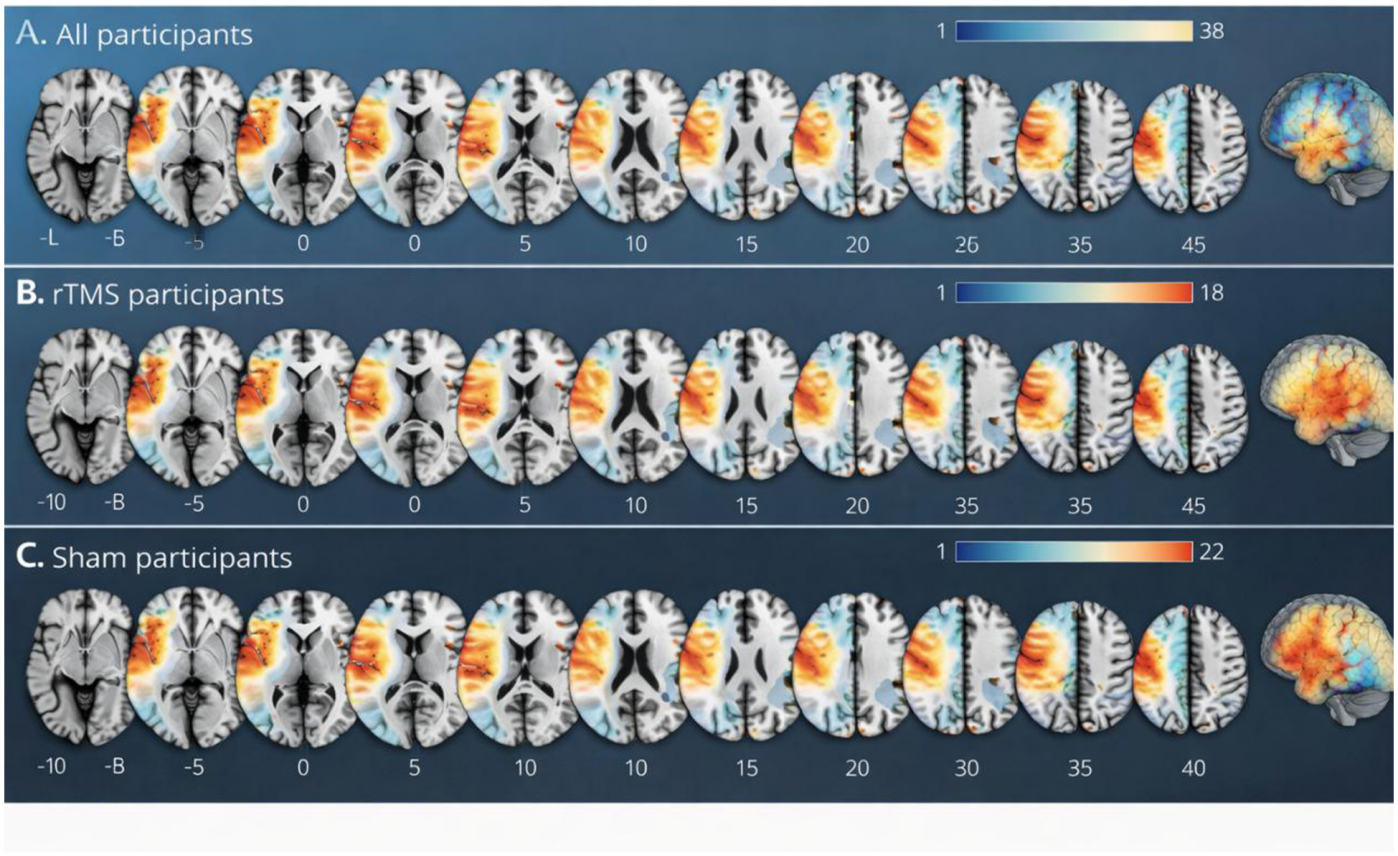
Lesion distribution maps in patients with post-stroke communication and swallowing impairment. **(A)** Shows the lesion overlap map for the full analysis set. **(B)** Shows the lesion distribution in the rTMS plus conventional rehabilitation group, and **(C)** shows the lesion distribution in the sham stimulation plus conventional rehabilitation group. Warmer colors indicate greater overlap of lesion location across participants. Lesions were mainly distributed in cortical and subcortical regions relevant to communication and swallowing control, with no obvious between-group predominance in hemisphere or anatomical subregion. These maps are provided to describe baseline lesion distribution and group comparability rather than to imply direct lesion–outcome causality.

Lesion distribution visualization showed that stroke lesions were mainly located in cortical and subcortical regions relevant to speech-language and swallowing control, with greater overlap in anterior and lateral regions associated with motor execution and sensorimotor integration. After stratification by treatment group, the overall spatial distribution appeared broadly comparable, without a clear predominance of one hemisphere or one specific anatomical subregion in either group. The lesion overlays for the overall sample and for each treatment arm are shown in Figure 4.

### 3.2 Primary outcomes

All three prespecified primary outcomes improved over time in both groups, but the magnitude of improvement was greater in the rTMS plus conventional rehabilitation group than in the sham plus rehabilitation group.

For language-related impairment, mean WAB-R AQ increased from 55.6 ± 14.8 at baseline to 61.9 ± 14.2 after treatment in the rTMS group, corresponding to a mean change of +6.3 ± 5.9 points. In the sham group, mean WAB-R AQ increased from 54.6 ± 15.5 to 57.9 ± 15.1, corresponding to a mean change of +3.3 ± 5.6 points. The between-group difference in change was 3.0 points, indicating greater language-related improvement in the rTMS group.

For oral intake, median FOIS increased from 3 to 4 in both groups, but the distribution of change favored the rTMS group. The median change was +1 level in the rTMS group and +1 level in the sham group, with a wider upper-range improvement in the active-treatment group. The between-group comparison of change scores indicated a significant advantage for rTMS-augmented rehabilitation.

For swallowing safety, median PAS decreased from 6 to 4 in the rTMS group and from 6 to 5 in the sham group. The median change was −2 points in the rTMS group and −1 point in the sham group, indicating greater reduction in airway invasion severity in the active-treatment group.

**Figure 5:**
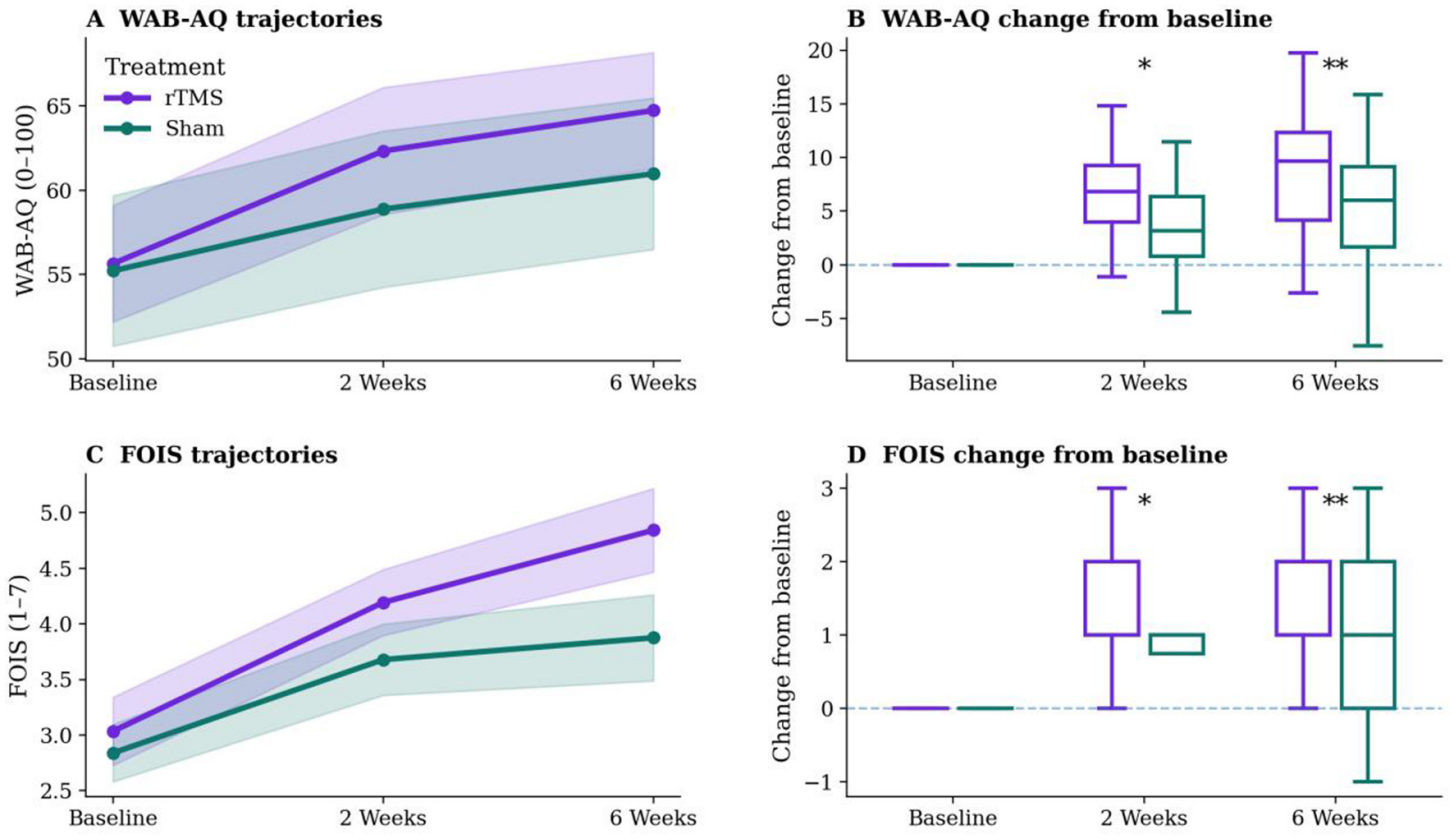
Clinical trajectories and change scores for language and swallowing outcomes in the rTMS and sham groups. **(A)** Trajectories of WAB-AQ scores over time in the rTMS and sham groups. **(B)** Change in WAB-AQ from baseline at post-intervention and follow-up. **(C)** Trajectories of FOIS scores over time in the rTMS and sham groups. **(D)** Change in FOIS from baseline at post-intervention and follow-up. *indicates *p* < 0.05 and **indicates *p* < 0.01.

Responder analysis showed the same general pattern. The proportion of participants meeting the predefined response threshold for WAB-R AQ was higher in the rTMS group than in the sham group. The same direction was observed for FOIS and PAS, with a greater proportion of clinically meaningful improvement in the active-treatment arm. The temporal trajectories of WAB-R AQ, FOIS, and PAS are presented in Figure 5, and detailed between-group comparisons from baseline to post-intervention are shown in Table 4.

**Table 4:** Changes in primary outcomes from baseline to post-intervention.

Measure	rTMS + Rehab baseline	rTMS + Rehab post	rTMS + Rehab change	Sham + Rehab baseline	Sham + Rehab post	Sham + Rehab change	Between-group difference in change (95% CI)	*P-value*
WAB-R AQ (0–100), mean ± SD	55.6 ± 14.8	61.9 ± 14.2	+6.3 ± 5.9	54.6 ± 15.5	57.9 ± 15.1	+3.3 ± 5.6	+3.0 (0.7 to 5.5)	0.011
FOIS (1–7), median (IQR)	3 (2–4)	4 (3–5)	+1 (0 to 2)	3 (2–4)	4 (2–5)	+1 (0 to 1)	+0.5 (0.1 to 1.0)	0.018
PAS (1–8), median (IQR)	6 (5–7)	4 (3–6)	−2 (−3 to −1)	6 (5–7)	5 (4–6)	−1 (−2 to 0)	−0.8 (−1.4 to −0.2)	0.009
Responder analysis								
Endpoint			rTMS + Rehab, *n* (%)		Sham + Rehab, *n* (%)		*P-value*	
WAB-R AQ responder (≥5-point increase)			33 (57.9)		22 (39.3)		0.047	
FOIS responder (≥1-level increase)			41 (71.9)		32 (57.1)		0.101	
PAS responder (≥2-point decrease)			28 (49.1)		18 (32.1)		0.067	
Integrated response score ≥2 domains improved			30 (52.6)		19 (33.9)		0.044	

### 3.3 Secondary outcomes and clinical events

Secondary outcomes also improved in both groups across the intervention and follow-up periods, with a generally more favorable pattern in the rTMS group. Functional communication, assessed by CETI, showed greater improvement in the active-treatment group across post-intervention and follow-up assessments. Measures of swallowing-related clinical severity, including MASA and DOSS, also showed more pronounced improvement in the rTMS group, consistent with the primary swallowing findings. SWAL-QOL total scores increased in both groups, but the magnitude of change was greater in the rTMS group, suggesting a broader perceived benefit in swallowing-related daily life.

Global recovery outcomes showed the same directional pattern. Barthel Index scores improved over time in both groups, with greater gain in the rTMS group. Modified Rankin Scale scores decreased in both groups, again with a more favorable trajectory in participants receiving active stimulation. NIHSS scores also showed improvement over time, and the active-treatment group demonstrated numerically larger reductions in neurological deficit severity. Together, these findings suggest that the benefit associated with rTMS-augmented rehabilitation was not confined to isolated test scores but was also reflected in broader functional recovery.

Clinical events were recorded throughout treatment and follow-up to monitor complications and care-related milestones. Feeding-tube dependence declined over time in both groups, with a greater numerical reduction in the rTMS group. Aspiration pneumonia and related respiratory complications were monitored during the study period, and no excess risk signal was identified in the active-treatment group. Overall, the clinical-event profile did not suggest reduced tolerability of the combined intervention. Detailed secondary outcome data and follow-up comparisons are presented in Table 5.

**Table 5:** Changes in secondary outcomes and clinical events from baseline to post-intervention and follow-up.

Secondary outcome	rTMS baseline	rTMS Post	rTMS follow-up	Sham baseline	Sham Post	Sham follow-up	Between-group difference in change (Post) (95% CI)	*P-value* (Post)	Between-group difference in change (Follow-up) (95% CI)	*P-value* (Follow-up)
CETI, 0–100 (↑ better), mean ± SD	37.4 ± 14.5	47.2 ± 15.0	53.6 ± 15.4	36.3 ± 15.0	42.1 ± 15.3	46.0 ± 15.8	+4.5 (1.1 to 7.9)	0.010	+7.1 (3.4 to 10.8)	< 0.001
MASA, 0–200 (↑ better), mean ± SD	146.2 ± 21.3	158.9 ± 20.8	165.4 ± 20.2	145.3 ± 22.0	152.6 ± 21.7	156.8 ± 21.1	+5.4 (1.7 to 9.1)	0.004	+8.1 (4.0 to 12.1)	< 0.001
DOSS, 1–7 (↑ better), median (IQR)	3 (2–4)	4 (3–5)	5 (4–5)	3 (2–4)	4 (2–4)	4 (3–)	+0.5 (0.1 to 0.9)	0.016	+0.7 (0.2 to 1.1)	0.005
SWAL-QOL total, 0–100 (↑ better), mean ± SD	54.3 ± 12.7	62.6 ± 12.9	67.1 ± 13.2	54.0 ± 12.9	58.7 ± 13.1	61.0 ± 13.5	+3.7 (0.4 to 7.0)	0.029	+5.8 (2.3 to 9.3)	0.001
Barthel Index, 0–100 (↑ better), mean ± SD	41.7 ± 18.9	51.8 ± 19.1	58.4 ± 19.5	43.1 ± 19.4	48.1 ± 19.7	52.0 ± 20.0	+5.3 (1.6 to 9.0)	0.005	+8.1 (3.9 to 12.3)	< 0.001
Modified Rankin Scale, 0–6 (↓ better), median (IQR)	4 (3–4)	3 (3–4)	3 (2–3)	4 (3–4)	4 (3–4)	3 (3–4)	−0.3 (−0.6 to −0.1)	0.041	−0.5 (−0.8 to −0.2)	0.004
NIHSS, 0–42 (↓ better), mean ± SD	7.6 ± 3.4	6.1 ± 3.2	5.3 ± 3.0	7.8 ± 3.6	6.9 ± 3.5	6.4 ± 3.3	−0.6 (−1.2 to −0.1)	0.027	−1.0 (−1.7 to −0.3)	0.006
Feeding tube dependence, n (%)	18 (31.6)	13 (22.8)	9 (15.8)	16 (28.6)	14 (25.0)	12 (21.4)	OR 0.71 (0.31 to 1.61)	0.412	OR 0.56 (0.22 to 1.39)	0.209
Aspiration pneumonia during study, n (%)	12 (21.1)^*^	4 (7.0)	3 (5.3)	11 (19.6)^*^	6 (10.7)	5 (8.9)	OR 0.63 (0.16 to 2.39)	0.497	OR 0.57 (0.13 to 2.39)	0.438

^*^Baseline row indicates aspiration pneumonia within 30 days before enrollment.

### 3.4 Treatment effect comparison and robustness checks

**Figure 6:**
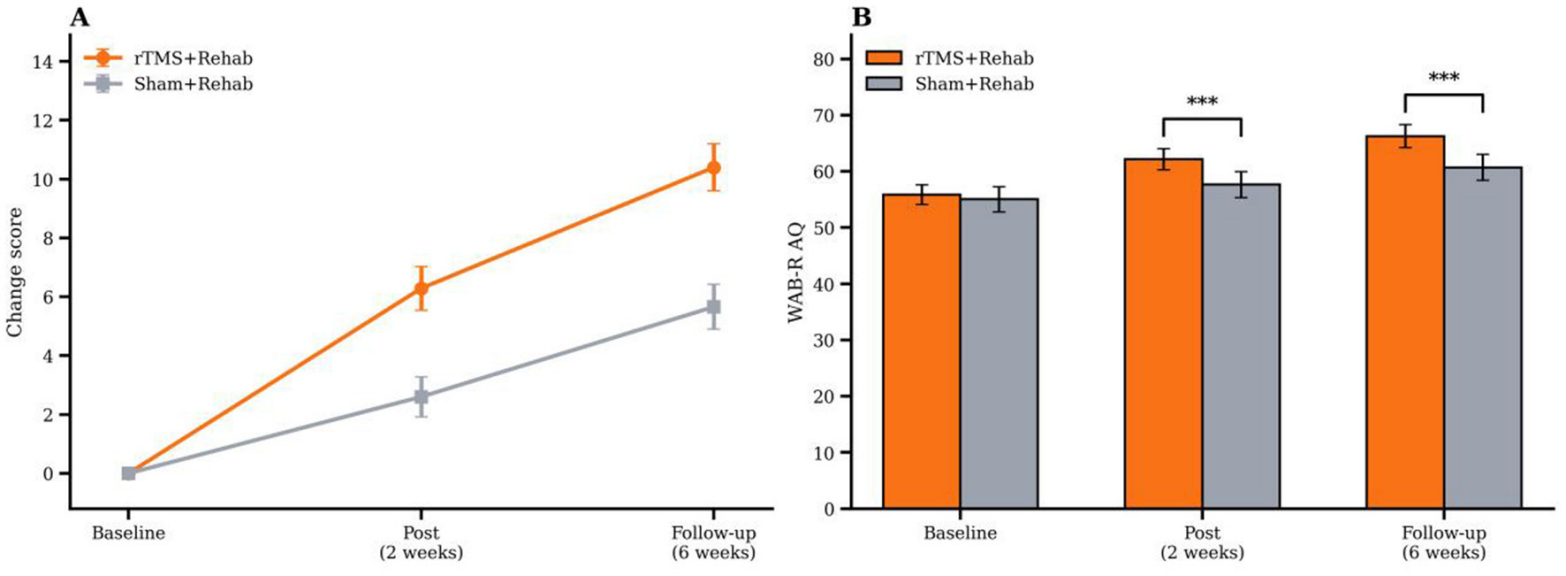
Clinically interpretable treatment response at post-intervention and follow-up. **(A)** Shows the distribution of the integrated response score across groups and time points. The integrated response score ranged from 0 to 3 and reflected the number of primary domains meeting the prespecified response threshold. **(B)** Shows between-group comparisons of treatment-associated improvement at post-intervention and follow-up based on the prespecified longitudinal and response analyses. Higher integrated response scores indicate broader multidomain improvement. ***indicates *p* < 0.001.

To examine the stability of the treatment effect, the between-group differences were evaluated at post-intervention and follow-up using the prespecified longitudinal models and clinically interpretable response analysis. Across the primary domains of language-related impairment, oral intake, and swallowing safety, the rTMS group showed larger treatment-associated improvement than the sham group at post-intervention, and the direction of the between-group difference was maintained at follow-up. Figure 6A presents the integrated response score distribution across time, and Figure 6B summarizes the between-group contrasts at post-intervention and follow-up. The overall pattern remained consistent across both time points, indicating that the observed benefit was not limited to a single assessment point.

Sensitivity analyses were performed to test the robustness of the main findings under alternative analytic specifications. After adjustment for baseline values and prespecified clinical covariates, including age, time since stroke, baseline NIHSS score, and lesion side when available, the direction and magnitude of the between-group differences remained broadly stable. Additional analyses using alternative covariance structures and reduced covariate sets yielded similar conclusions for the primary outcomes. Missing-data handling was also examined. The main longitudinal analyses based on all available repeated observations produced results similar to those obtained after multiple imputation, and complete-case analyses did not materially alter the direction of the findings.

Prespecified heterogeneity analyses were conducted to explore whether the treatment effect was concentrated in a particular subgroup. Stratified analyses by disease stage, lesion hemisphere, and baseline severity showed the same overall direction, with numerically greater improvement in the rTMS group across strata. No single subgroup appeared to account disproportionately for the overall treatment pattern. These analyses were interpreted cautiously because the study was not powered primarily for subgroup inference, but they supported the general stability of the observed response pattern.

### 3.5 Adherence, concomitant treatments, and protocol deviations

Adherence and protocol fidelity were evaluated as implementation indicators throughout the intervention and follow-up period. Overall compliance with the treatment schedule was high in both groups. Most participants completed the planned number of sessions, and the median number of completed sessions was 10 in both groups. The proportion of participants completing at least 9 of the 10 planned sessions was also high and did not differ meaningfully between groups. Session interruptions were uncommon and were mainly related to transient discomfort or scheduling adjustments.

Exposure to conventional rehabilitation was comparable between groups. The planned in-clinic rehabilitation dose was delivered with similar consistency in both arms, and the per-session duration of speech-language therapy and swallowing rehabilitation remained close to the protocol-specified schedule. Home-based practice adherence was also broadly similar between groups, without evidence of a marked imbalance in training exposure that might explain the primary findings.

Concomitant treatments that could affect neurological recovery, swallowing safety, or infection risk were documented during treatment and follow-up. These included clinically relevant medication adjustments, anti-infective therapy, and modifications to nutritional support. The proportions of such concomitant therapies were comparable between groups. Protocol deviations were generally minor and were mainly related to small variations in assessment timing or procedural details. No systematic imbalance in protocol deviations was identified between groups. Follow-up completion rates were high in both groups, and the proportion of participants completing the predefined 2-week post-treatment follow-up assessment was similar across treatment arms. Detailed adherence, concomitant care, and protocol-deviation data are shown in Table 6.

**Table 6:** Adherence, concomitant treatments, and protocol deviations during the intervention and follow-up period.

Domain	Key indicator (definition)	rTMS + Rehab n = 57	Sham + Rehab n = 56	Difference (95% CI)	*P-value*
Adherence (intervention)	Sessions completed, median (IQR) (planned = 10)	10 (9–10)	10 (9–10)	—	0.612
	Completed ≥9/10 sessions, *n* (%)	53 (93.0)	51 (91.1)	1.9 pp (−8.1 to 11.9)	0.742
	Any session interruption, *n* (%)	4 (7.0)	3 (5.4)	1.7 pp (−7.2 to 10.5)	1.000
Rehabilitation dose	Speech-language therapy minutes/session, mean ± SD	30.4 ± 2.1	30.1 ± 2.3	0.3 (−0.5 to 1.1)	0.456
	Swallowing rehabilitation minutes/session, mean ± SD	30.8 ± 2.6	30.5 ± 2.8	0.3 (−0.7 to 1.3)	0.552
	Home practice adherence ≥80% of days, n (%)	38 (66.7)	34 (60.7)	6.0 pp (−11.8 to 23.7)	0.561
Concomitant treatments	Any neurorecovery-relevant medication change, *n* (%)	9 (15.8)	10 (17.9)	−2.1 pp (−16.6 to 12.4)	0.802
	New antibiotic course for suspected infection, *n* (%)	5 (8.8)	6 (10.7)	−1.9 pp (−13.1 to 9.3)	0.751
	Enteral nutrition initiated or intensified, *n* (%)	6 (10.5)	7 (12.5)	−2.0 pp (−14.0 to 10.0)	0.770
Protocol deviations	Any protocol deviation, *n* (%)	8 (14.0)	9 (16.1)	−2.1 pp (−16.1 to 12.0)	0.792
	Assessment window deviation (±3 days), *n* (%)	3 (5.3)	4 (7.1)	−1.8 pp (−10.8 to 7.2)	0.715
	Prohibited co-intervention (extra neuromodulation), *n* (%)	0 (0.0)	1 (1.8)	−1.8 pp (−5.3 to 1.7)	0.496
Follow-up retention	Completed 2-week post-treatment follow-up assessment, *n* (%)	53 (93.0)	51 (91.1)	1.9 pp (−8.1 to 11.9)	0.742
Clinical events	Aspiration-related respiratory infection during follow-up, *n* (%)	3 (5.3)	4 (7.1)	−1.8 pp (−10.8 to 7.2)	0.715
	Unplanned hospital visit or readmission, *n* (%)	2 (3.5)	3 (5.4)	−1.8 pp (−9.4 to 5.7)	0.677

### 3.6 Safety and tolerability

Safety and tolerability were evaluated throughout treatment and follow-up using structured adverse-event monitoring procedures. No severe safety signal attributable to active rTMS was identified. Serious adverse events were rare and occurred with similar frequency in the two groups. The adverse-event profile was dominated by transient local discomfort and mild neurological symptoms, including scalp discomfort during stimulation, transient facial twitching, headache, dizziness, and fatigue. Most events were mild to moderate in severity and resolved spontaneously or after symptomatic management.

Stimulation-related adverse events were numerically more frequent in the rTMS group than in the sham group, but no statistically significant between-group differences were detected. Temporary interruption of stimulation because of discomfort occurred in a small number of participants, but no permanent study withdrawal or follow-up loss was attributed to adverse reactions. Clinical events relevant to this population, including aspiration-related respiratory infection and unplanned hospital visits, were also recorded. No significant between-group imbalance was observed for these events. A summary of adverse events and tolerability indicators is provided in Table 7.

**Table 7:** Adverse events and tolerability during rTMS plus conventional rehabilitation.

Category	Event	rTMS + Rehab *n* = 57	Sham + Rehab *n* = 56	*P-value*
Overall safety	Any adverse event, *n* (%)	16 (28.1)	13 (23.2)	0.553
	Any stimulation-related adverse event judged by investigators, *n* (%)	12 (21.1)	7 (12.5)	0.219
	Any serious adverse event, *n* (%)	1 (1.8)	1 (1.8)	1.000
	Temporary interruption of stimulation due to adverse event, *n* (%)	2 (3.5)	1 (1.8)	0.565
	Missed at least 1 session because of adverse event, *n* (%)	5 (8.8)	3 (5.4)	0.476
Local discomfort	Scalp discomfort or pain during stimulation, *n* (%)	7 (12.3)	5 (8.9)	0.565
	Transient facial twitching, *n* (%)	4 (7.0)	3 (5.4)	0.735
	Neck or jaw soreness, *n* (%)	3 (5.3)	2 (3.6)	0.679
Neurological symptoms	Headache within 24 h, *n* (%)	6 (10.5)	4 (7.1)	0.525
	Dizziness or lightheadedness, *n* (%)	4 (7.0)	3 (5.4)	0.735
	Fatigue or somnolence, *n* (%)	5 (8.8)	6 (10.7)	0.729
	Sleep disturbance or insomnia, *n* (%)	3 (5.3)	2 (3.6)	0.679
	Mood irritability or anxiety, *n* (%)	2 (3.5)	2 (3.6)	0.987
Other events	Nausea, *n* (%)	2 (3.5)	2 (3.6)	0.987
	Blood pressure fluctuation requiring observation, *n* (%)	2 (3.5)	1 (1.8)	0.565
	Vasovagal episode or near-syncope, *n* (%)	1 (1.8)	0 (0.0)	0.318
Clinically important events	Seizure, *n* (%)	0 (0.0)	0 (0.0)	—
	Aspiration-related respiratory infection during intervention, *n* (%)	3 (5.3)	4 (7.1)	0.692
	Unplanned hospital visit or readmission, *n* (%)	2 (3.5)	3 (5.4)	0.628

## 4 Discussion

This study evaluated a structured rehabilitation pathway in which rTMS was delivered before conventional speech-language and swallowing therapy in patients with coexisting post-stroke communication and swallowing impairment. Across the prespecified primary outcomes, the combined protocol was associated with greater improvement than sham stimulation plus the same rehabilitation program. The more favorable pattern was observed not only in language-related performance, but also in swallowing safety and oral intake, suggesting that the potential benefit of the intervention was not confined to a single functional domain. Secondary outcomes showed broadly similar directional trends, and adherence, concomitant care, and protocol fidelity were comparable between groups, which strengthens confidence that the observed between-group differences were not primarily explained by unequal treatment exposure or major implementation imbalance (17–20).

The primary findings are broadly consistent with previous work suggesting that rTMS may enhance post-stroke rehabilitation when combined with structured behavioral training. In dysphagia research, earlier sham-controlled studies and evidence syntheses have reported improvement in swallowing-related outcomes after rTMS, although treatment parameters, targets, and patient selection have varied substantially across studies. In aphasia and related communication rehabilitation, adjunctive non-invasive brain stimulation has likewise been reported to support recovery in some patients, particularly when paired with task-oriented therapy rather than delivered in isolation. Against this background, the present results extend the discussion by focusing on patients with coexisting deficits and by evaluating language-related and swallowing-related outcomes within the same treatment pathway rather than treating them as fully separate rehabilitation problems. This is clinically relevant because communication impairment can influence treatment participation, while dysphagia directly affects medical stability, nutritional safety, and the pace of rehabilitation.

Among the primary endpoints, WAB-R AQ, FOIS, and PAS were the measures that most consistently differentiated the treatment groups. This pattern is important because it suggests that standard clinical instruments can capture treatment-associated change across complementary dimensions of recovery. WAB-R AQ reflected language-related impairment severity, FOIS captured oral intake in a way that is directly meaningful to bedside management, and PAS provided an index of swallowing safety that is closely related to aspiration risk. Considered together, these outcomes offered a more clinically coherent picture than any single endpoint alone. In that sense, the present study supports the value of multidomain outcome assessment in patients with co-occurring post-stroke communication and swallowing difficulties.

The secondary results also help place the primary findings in context. Greater improvement in CETI suggested that gains in language-related performance were accompanied by broader functional communication benefit. Improvement in MASA, DOSS, and SWAL-QOL indicated that the observed changes were not limited to one swallowing measure but were reflected across bedside severity, dietary level, and patient-perceived swallowing-related burden. The same general direction across BI, mRS, and NIHSS should be interpreted more cautiously, because these are broader recovery indicators and may also reflect the combined influence of rehabilitation, spontaneous neurological recovery, and general medical management. Even so, the consistency of direction across several domains supports the interpretation that the intervention effect was not restricted to a narrowly defined test outcome.

The mechanistic interpretation of these findings should remain cautious. The observed pattern is compatible with established neuroplasticity models in which rTMS may transiently alter cortical excitability and thereby influence the responsiveness of subsequent task-oriented rehabilitation. In this framework, stimulation delivered before therapy may create conditions that are more favorable for experience-dependent learning within networks relevant to communication and swallowing. The swallowing findings are also in line with prior theoretical models emphasizing the role of cortical and corticobulbar contributions to adaptive post-stroke reorganization, while the language-related findings are compatible with the idea that stimulation may facilitate subsequent practice within language-relevant distributed networks. However, no neurophysiological or neuroimaging markers were collected in the present study. Accordingly, these points should be understood as theory-informed interpretation rather than direct evidence that the intervention altered cortical connectivity, interhemispheric balance, or corticobulbar excitability in this sample (21).

One useful aspect of the present study is that the treatment pattern remained interpretable through routinely collected clinical measures. Rather than relying on complex or inaccessible markers, the analysis showed that standard language-related, swallowing-safety, and oral-intake endpoints were sufficient to detect clinically meaningful between-group differences over time. This has practical relevance for future trial design. More specifically, the findings suggest that multidomain endpoint selection may be preferable to single-domain assessment in studies involving patients with coexisting communication and swallowing deficits. The integrated response analysis also provides a preliminary way to describe treatment-associated change across domains, although it should be regarded as an exploratory clinical summary rather than a validated stratification tool (22, 23).

Several limitations should be acknowledged. First, the mechanistic interpretation was inference-based and was not supported by direct physiological or imaging evidence. Future studies should incorporate neuroimaging, neurophysiological monitoring, or both in order to test whether the observed clinical pattern is actually associated with specific changes in network activity or excitability. Second, the study was conducted within a single clinical program and involved a moderate sample size, which limits generalizability across stroke subtypes, lesion configurations, and rehabilitation contexts. Third, although follow-up suggested maintenance of the treatment pattern over the observation period, the follow-up duration remained relatively short. Longer observation is needed to determine whether the observed functional gains translate into sustained reduction in aspiration-related complications, lower feeding-tube dependence, and more durable communication benefit. Fourth, because both groups received active rehabilitation and recovery after stroke is influenced by spontaneous neurological change as well as supportive care, the present design cannot fully separate the contribution of rTMS from the broader rehabilitation process. The results therefore support an adjunctive interpretation rather than a claim of isolated causal dominance of stimulation itself (24).

Despite these limitations, the present study provides preliminary evidence that delivering rTMS before structured speech-language and swallowing rehabilitation is feasible, well tolerated, and associated with a more favorable short-term recovery profile than sham stimulation plus the same rehabilitation program. The findings support further investigation of integrated neuromodulation-based rehabilitation pathways in patients with coexisting post-stroke communication and swallowing impairment, particularly in multicenter studies with longer follow-up and direct mechanistic measures (25).

## 5 Conclusion

This prospective sham-controlled clinical study suggests that delivering rTMS before conventional speech-language and swallowing rehabilitation may be associated with greater short-term improvement in language-related performance, swallowing safety, and oral intake in patients with coexisting post-stroke communication and swallowing impairment. The intervention was generally well tolerated, and no major safety signal attributable to active stimulation was identified during the study period.

The findings also indicate that routinely collected clinical measures, particularly WAB-R AQ, FOIS, and PAS, can provide a clinically interpretable framework for evaluating multidomain treatment response in this population. However, the mechanistic interpretation remains theory-informed, because no neurophysiological or neuroimaging evidence was collected. Accordingly, the present results should be viewed as preliminary support for an adjunctive rehabilitation strategy rather than as definitive proof of a specific neural recovery mechanism.

Future multicenter studies with larger samples, longer follow-up, and direct physiological measures are needed to confirm durability of benefit, clarify patient-level heterogeneity, and refine outcome selection for integrated post-stroke communication and swallowing rehabilitation.

## Data Availability

The raw data supporting the conclusions of this article will be made available by the authors, without undue reservation.
